# Induction of Oxidative DNA Damage in Bovine Herpesvirus 1 Infected Bovine Kidney Cells (MDBK Cells) and Human Tumor Cells (A549 Cells and U2OS Cells)

**DOI:** 10.3390/v10080393

**Published:** 2018-07-26

**Authors:** Liqian Zhu, Xiaotian Fu, Chen Yuan, Xinyi Jiang, Gaiping Zhang

**Affiliations:** 1 College of Veterinary Medicine, Yangzhou University, Yangzhou 225009, China; fxt950828@163.com (X.F.); yuanchen060624@163.com (C.Y.); jxy0492@163.com (X.J.); 2 Jiangsu Co-innovation Center for Prevention and Control of Important Animal Infectious Diseases and Zoonoses, Yangzhou 225009, China; 3 Key Laboratory for Animal Immunology of the Ministry of Agriculture, Henan Provincial Key Laboratory of Animal Immunology, Henan Academy of Agricultural Sciences, Zhengzhou 450002, China; 4 College of Animal Science and Veterinary Medicine, Henan Agricultural University, Zhengzhou 450000, China

**Keywords:** BoHV-1, DNA damage, OGG-1, ROS, comet assay, tumor

## Abstract

Bovine herpesvirus 1 (BoHV-1) is an important pathogen of cattle that causes lesions in mucosal surfaces, genital tracts and nervous systems. As a novel oncolytic virus, BoHV-1 infects and kills numerous human tumor cells. However, the mechanisms underlying the virus-induced cell damages are not fully understood. In this study, we demonstrated that virus infection of MDBK cells induced high levels of DNA damage, because the percentage of comet tail DNA (tailDNA%) determined by comet assay, a direct indicator of DNA damage, and the levels of 8-hydroxyguanine (8-oxoG) production, an oxidative DNA damage marker, consistently increased following the virus infection. The expression of 8-oxoguanine DNA glycosylase (OGG-1), an enzyme responsible for the excision of 8-oxoG, was significantly decreased due to the virus infection, which corroborated with the finding that BoHV-1 infection stimulated 8-oxoG production. Furthermore, the virus replication in human tumor cells such as in A549 cells and U2OS cells also induced DNA damage. Chemical inhibition of reactive oxidative species (ROS) production by either ROS scavenger *N*-Acetyl-l-cysteine or NOX inhibitor diphenylene iodonium (DPI) significantly decreased the levels of tailDNA%, suggesting the involvement of ROS in the virus induced DNA lesions. Collectively, these results indicated that BoHV-1 infection of these cells elicits oxidative DNA damages, providing a perspective in understanding the mechanisms by which the virus induces cell death in both native host cells and human tumor cells.

## 1. Introduction 

Bovine herpesvirus-1 (BoHV-1) is a large enveloped double stranded DNA virus. Together with herpes simplex 1 and 2 (HSV-1, HSV-2) and varicella zoster virus (VZV), BoHV-1 belongs to the family *Herpesviridae* and the subfamily *Alphaherpesvirinae* [1,2]. BoHV-1 is a widespread cattle pathogen causing severe respiratory infection, conjunctivitis, vaginitis, balanoposthitis, abortion, and encephalitis [2,3]. Acute virus infection causes lesions on mucosal surfaces, corpus luteum, and the nervous system followed by the establishment of life-long latency primarily in trigeminal ganglia [3,4]. Due to immune suppression and mucosal lesions by the virus infection, secondary infection by diverse bacteria tends to occur, and consequently causes bovine respiratory disease complex (BRDC), the costliest disease for cattle [1,5]. In view of the fact that the virus induced lesions in the respiratory tract, productive tract and nerve system are associated with diseases outcome, a better understanding of the molecular basis of virus-induced cell damage would be helpful to learn its pathogenesis.

Oncolytic viruses selectively replicate in and kill tumor cells while sparing normal cells [6]. Oncolytic virotherapy seems to represent a promising alternative in the light of the limited efficacy and severe side effects in conventional cancer therapeutics [7,8]. BoHV-1 is able to infect and kill a variety of immortalized and transformed human cell types, including human breast tumor cell lines MCF-10A cells, HME-1 cells and MDA-MB-468 cells, prostate tumor cell line RWPE-1 cells, A549 lung carcinoma cells, and bone osteosarcoma epithelial cells U2OS [9,10]. Despite the fact that BoHV-1 shares some features with HSV-1, BoHV-1 has a restricted host range, and is unable to productively infect humans. BoHV-1 may selectively replicate in tumor cells by exploiting the biochemical differences between normal and tumor cells [11]. Moreover, BoHV-1 infection of human tumor cells fails to elicit interferon (IFN) production, and the oncolytic effects are not correlated with type I IFN signaling [10], which may be a benefit for escaping the eradication effects of the IFN-mediated virus, in vivo. Interestingly, using a spontaneous and genetically engineered breast cancer murine model, it has been revealed that BoHV-1 could kill bulk breast cancer cells and cancer-initiating cells from luminal and basal subtypes [12], which highlighted the efficacy of BoHV-1 oncolytic effects, in vivo. Given the safety to human beings along with prominent efficacy, BoHV-1 is an attractive candidate for virotherapy to combat diverse cancers. However, the mechanisms by which BoHV-1 elicits cell damages in human tumor cells are not yet completely known.

Reactive oxidative species (ROS) such as superoxide, hydrogen peroxide (H_2_O_2_), peroxynitrite (OONO−) and hydroxyl radical (OH) are generated ubiquitously by all mammalian cells. In physiological concentration, ROS are important for normal biologic processes, whereas excessive ROS can damage cell components such as lipids, proteins, nucleic acids and carbohydrates [13,14]. HSV-1 infection elevates cellular ROS levlels in murine microglial cells, which is associated with production of proinflammatory cytokines and neural cell damage [15,16]. ROS overproduction and different cell death forms were induced in neuronal and glial-derived tumor cells following BoHV-1 and BoHV-5 infection [17]. These studies unanimously addressed the importance of ROS in herpesvirus induced cell death. Furthermore, treatment of U251T3 glioma cells(a tumor cells) with FDA-approved proteasome inhibitor bortezomib along with an oncolytic herpes simplex virus-1 (oHSV) expressing GMCSF promotes ROS production and necroptotic cell death [18], adding support to the potential role of ROS played in herpesviruses infection-induced cell death. 

DNA damage gives rise to mutations and chromosomal abnormalities, and consequently induces cell death by diverse mechanisms, including but not limited to, the activation of caspase-dependent and -independent apoptosis machines [19,20], the activation of poly(ADP-ribose) polymerase-1 (PARP-1) to cause necrotic cell death [21,22], and the activation of autophagic cell death pathways [23]. Since DNA is vulnerable to the insult of ROS [24], it is reasonable to speculate that overprodution of ROS due to virus infection may lead to DNA damage. We hypothesized that BoHV-1 infection induced oxidative DNA damage, which potentially contributed to the virus-induced cell damage in diverse cell types including human tumor cells.

In this study, we initially used MDBK cells to explore the impact of BoHV-1 infection on DNA damage. By detection of tailDNA% and 8-oxoG, two canonical indicators for DNA damage, we showed that the level of DNA damage was increased following BoHV-1 infection. And the increased DNA damage was closely associated with overproduced ROS. Importantly, oxidative DNA damage was induced during the infection of human tumor cells, including in A549 cells and U2OS cells. Collectively, for the first time, we provide evidence that BoHV-1 infection elicited oxidative DNA damage, a potential mechanism by which the virus induced cell damage.

## 2. Materials and Methods 

### 2.1. Cells and Virus

A549 cells U2OS cells (Gifts from Dr. Renjin Chen, Xuzhou Medical University, Xuzhou, China) were maintained in DMEM (Gibco, Thermo Fisher Scientific, Waltham, MA, USA) supplemented with 10% fetal bovine serum (HyClone Laboratories, Logan, UT, USA). MDBK cells (Purchased from Chinese model culture preservation center, Shanghai, China) were maintained in DMEM supplemented with 10% horse serum (HyClone Laboratories, Logan, UT, USA). BoHV-1 (Colorado1 stain) was propagated in MDBK cells. Aliquots of virus stocks were stored at −70 °C until use. 

### 2.2. Antibodies and Reagents

8-oxoG DNA lesion antibody (#sc-130914) was provided by Santa Cruz Biotechnology (Dallas, TX, USA). Fluorescein isothiocyanate labeled goat anti-mouse IgG (#BA1101) was purchased from Beijing Biosynthesis Biotechnology Co., Ltd. (Beijing, China). A goat anti-BoHV-1 serum was purchased from VMDR Inc. (Gandhinagar, India, #PAB-IBR). Donkey anti-goat IgG H&L (HRP) (Ca#ab97110) was ordered from abcam (Cambridge, MA, USA). ROS scavenger *N*-Acetyl-l-cysteine (#A7250), NOX inhibitor diphenylene iodonium (DPI) (#D2926), phosphonoacetic acid (PAA) (Cat #284270), ROS fluorescence indicator 2′,7′–dichlorodihydrofluorescein diacetate (H2DCFDA) (#D6883) was provided by Sigma-Aldrich (St. Louis, MO, USA). Propidium Iodide (#ST511) was ordered from Beyotime Biotechnology (Shanghai, China). BoHV-1 VP16 antibody is kindly provided by Prof. Vikram Misra at the University of Saskatchewan [25].

### 2.3. Immunofluorescence Assay

MDBK cells seeded in 6-well plates were mock infected or infected with BoHV-1 at an MOI of 0.1 for 12, 24 and 36 h. Cells were fixed in 4% paraformaldehyde in PBS (pH 7.4) for 10 min at room temperature, permeabilized with 0.25% Triton X-100 in PBS (pH 7.4) for 10 min at room temperature, blocked with 1% BSA in PBST (PBS+ 0.1% Tween20) for 30 min at room temperature, and incubated with anti-8-oxoG DNA lesion antibody in 1% BSA in PBST for 12 h at 4 °C. After three washes, cells were incubated with FITC labeled goat anti-mouse IgG (H + L) for 1 h in the dark. After three washes with PBS, images were obtained by fluorescent microscopy (Olympus BX-51; Olympus, Tokyo, Japan).

### 2.4. Western Blotting Analysis

MDBK cells, A549 cells and U2OS cells in 60 mm dishes were infected with BoHV-1 at MOI of 0.1 for the designated times, in the presence of 2% horse or fetal bovine serum. Cells were lysed with RIPA buffer (1 × PBS, 1% NP-40, 0.5% sodium deoxycholate, 0.1% SDS) supplemented with protease inhibitor cocktail (Roche, Basel, Switzerland). Cell lysates were clarified by centrifugation at 13,000 rpm for 10 min. The clarified supernatant was subjected to Western blotting analysis using the antibodies against VP16 (1:3000 dilution), β-actin (1:1000 dilution) or GAPDH (1:1000 dilution), and BoHV-1 viron (1:6000 dilution). The intensity of detected protein bands were quantitatively analyzed with free software image J (https://imagej.nih.gov/ij/download.html). 

### 2.5. Quantification of mRNA by qRT-PCR

MDBK cells in 60 mm dishes were infected with BoHV-1 using an MOI of 0.1. At 24 and 36 h after infection total RNA was purified with TRIzol LS Reagent (Ambion, Thermo Fisher Scientific, Waltham, MA, USA, cat# 10296010) following the manufacturers’ instructions. Freshly prepared RNA (1 μg) was used as a template for the synthesis of first-strand cDNA with commercial random hexamer primers for viral mRNA detection using Thermoscript™ RT-PCR system Kit (Invitrogen, Carlsbad, CA, USA, cat#11146-024). The cDNA products were used as templates for qPCR to measure levels of 8-Oxoguanine DNA glycosylase (OGG1) mRNA with the following gene-specific primers for forward: 5′-TGGTTCTGGCTTCTGGACAGT-3′, reverse: 5′-GCCAACCCTGCCCTTGT-3′. Meanwhile, glyceraldehyde-3-phosphate dehydrogenase (GAPDH) mRNA levels were detected with the following primers: forward: 5′-CCATGGAGAAGGCTGGGG-3′. reverse: 5′-AAGTTGTCATGGATGACC-3′. qPCR was carried out using the ABI 7500 fast real-time system (Applied Biosystems, Foster City, CA, USA). Analysis of GAPDH mRNA was used as an internal control to normalize gene expression. The data was analyzed using the equation 2^−ΔΔCT^ method.

### 2.6. Comet Assay

DNA damage was evaluated through alkaline comet assay (single cell gel electrophoresis) according to the described method with modification [26]. In brief, to examine virus-induced DNA damage over a time-course of infection, MDBK cells, A549 cells and U2OS cells in 24-well plates were mock infected with supernatant of cell cultures that were made by frozen-thawing and subsequent centrifugation using a procedure similar to the generation of virus stocks, or infected with virus for designated time duration. To determine the effects of ROS had on DNA damage, the cells pretreated with DMSO control, NAC or DPI at indicated concentrations for 1 h, were infected with BoHV-1 (MOI = 0.1) for indicated time lengths. At the designated time points following infection, cells were suspended in low melting agarose placed on slides coated with 1% normal melting agarose, and low melting agarose was then added as the top layer. Cells were lysed in cold (4 °C) lysis buffer (2.5 M NaCl, 100 mM Na2EDTA, 10 mM Tris, 1% Triton X, and 10% DMSO, pH 10.0) for 1 h. The slides were subjected to horizontal gel electrophoresis in cold (4 °C) alkaline electrophoresis buffer (300 mM NaOH, 1 mM Na2EDTA, pH 12.5) at 25 V and 300 mA for a time duration ranged from 20 min to 40 min depending on cell types. The slides were then soaked twice with neutralization buffer (0.4 M Trizma base, pH 7.5, 4 °C) for 10 min and air-dried. DNA was stained with PI (20 μg/mL) and quickly analyzed using a fluorescence microscope. About three hundred randomly captured cells from each sample were analyzed using CASP software (University of Wroclaw, Wrocław, Poland). DNA percentage in the tail (tailDNA%) were used as the metric for DNA migration [27].

### 2.7. Cellular ROS Assay

MDBK cells, A549 cells and U2OS cells in 24-well plates were pretreated with solvent DMSO, NAC or DPI at indicated concentrations for 1 h, then infected with BoHV-1 (MOI = 0.1) in the presence of a corresponding inhibitor for 1 h. The uninfected control was treated with cell lysates from uninfected MDBK cells. After washing with PBS for three times, fresh medium containing inhibitors were added. At 24 h after infection, the cells were washed with PBS and exposed to ROS fluorescence indicator H2DCFDA (50 μM) for 30 min at 37 °C. The reaction mixture was then replaced with PBS, and images were acquired under a fluorescence microscope, the fluorescence intensity of cellular ROS was quantified with software Image-pro Plus 6.

### 2.8. Cell Viability Assay 

Cell viability was assessed by Trypan blue exclusion test, as described by Fiorito et al. [28,29], with modification. In brief, MDBK cells, U2OS cells and A549 cells with or without infection in 24-well plates were treated with or without chemicals at indicated concentrations for designated time durations. Then the cells were collected by trypsinization, and an aliquot of the cell suspension was mixed with an equal volume of 0.4% Trypan-blue (0.4%) (Bio-Rad, Hercules, CA, USA, #1450021). After 10 min, cells were counted using a Burker chambre under a light microscope. The percentage of cell viability in the chemical treatment groups was calculated by normalization of the number of live cells to that in the control samples. The value of cell viability in the control was arbitrarily set to 100%.

## 3. Results

### 3.1. BoHV-1 Infection of MDBK Cells Results in Increased Levels of DNA Damage

**Figure 1:**
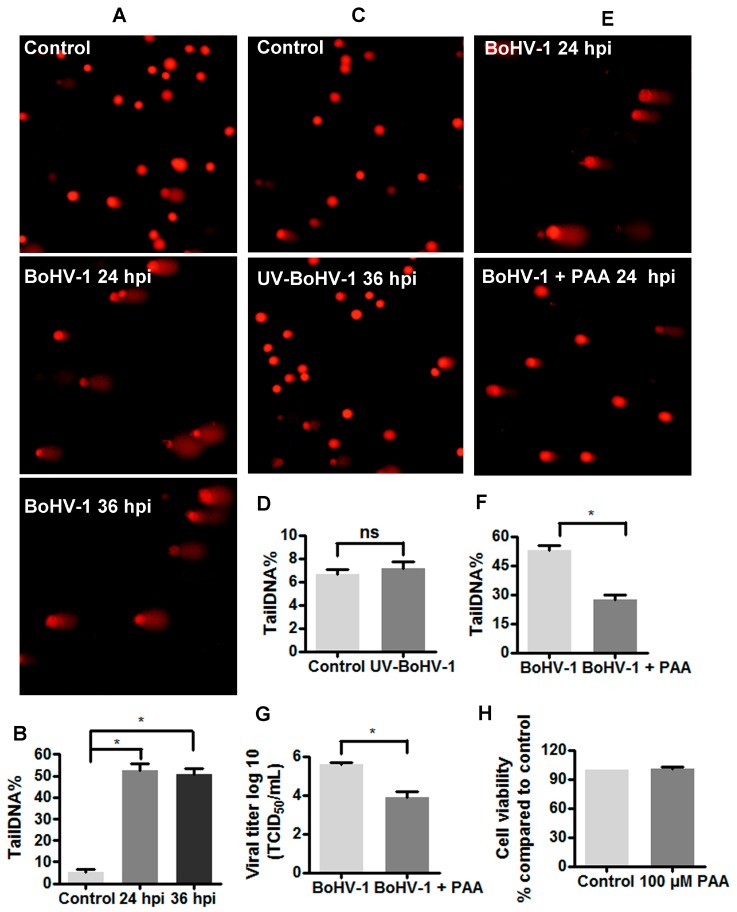
The detection of DNA damage in BoHV-1 infected MDBK cells. (**A**) MDBK cells were infected with BoHV-1 (MOI = 0.1), and at indicated time points the cell cultures were collected to measure DNA damage in individual cells with comet assay. The images were acquired under a fluorescence microscope. Results were of three independent experiments (Magnification ×200). (**B**) Three hundred cells randomly selected from each sample were used for the calculation of tailDNA% by software CASP. Values represented three independent experiments. *, significant differences (*P* < 0.05) in tailDNA%, as determined by a Student *t* test. (**C**) MDBK cells were infected with UV-inactivated BoHV-1 (MOI = 0.1). After infection for 36 h, the cells were collected and subjected to comet assay. The images were acquired under a fluorescence microscope. Results were of three independent experiments (Magnification ×200). (**D**,**F**) Three hundred cells randomly selected from each sample were used for the calculation of TailDNA% by software CASP. Values represented three independent experiments. *, significant differences (*P* < 0.05) in tailDNA%, as determined by a Student *t* test. (**E**) MDBK cells were infected with BoHV-1 (MOI = 0.1), along with phosphonoacetic acid (PAA) treatment (100 μM). At 24 h after infection, comet assay was performed to detect DNA damage in individual cells, the images were acquired under a fluorescence microscope. Results were of three independent experiments (Magnification ×200). (**G**) MDBK cells in 24-wells plates were infected with BoHV-1 (MOI = 0.1) and treated with PAA treatment (100 μM) or DMSO control for 24 h. The virus titer was determined in MDBK cells. Data represent three independent experiments. Significance was assessed with the Student *t* test (*, *P* < 0.05). (**H**) The cytotoxicity of PAA treated MDBK cells for 24 h was analyzed by Trypan-blue exclusion test. Data represent three independent experiments.

The alkaline version of comet assay is a sensitive and widely used method to assess DNA damage in individual cells [26,30]. As the frequency of DNA breaks increases, so does the fraction of the DNA extending towards the anode, forming the comet tail after electrophoresis in alkali [31]. MDBK cells are bovine kidney cells that are permissive to BoHV-1infection, and are widely used for studying BoHV-1 biology and pathogenesis. Initial studies examined the effects of BoHV-1 productive infection on DNA damage in MDBK cells using comet assay. As expected, a subset of MDBK cells with clear comet tails were readily detected at high frequency following the virus infection for either 24 h or 36 h, but not in the mock infected control (Figure 1A). The extent of DNA damage [strand breaks (SBs)] is best expressed as the percentage of DNA fluorescence in the tail (tailDNA%), which can be calculated using software. When MDBK cells were infected for 24 h and 36 h, the levels of tailDNA% were consistently increased to approximately 50%, while the steady state levels of tailDNA% in the mock infected control was ~5% (Figure 1B). The levels of tailDNA% were increased approximately ten fold by the virus infection, suggesting that virus infection may induce DNA damage. 

Ultraviolet light-inactivated viruses are replication deficient, because it could bind to the virus receptors and enter the cells, but not express viral genes [32,33]. To further understand how BoHV-1 infection induced DNA damage, MDBK cells were exposed to UV-inactivated virus for 36 h and subjected to comet assay. As in the mock infected controls, only a few cells with comet tails were detected from the cells exposed to UV-inactivated virus (Figure 1C). Infection by UV-inactivated virus could not significantly increase tailDNA%, relative to that in uninfected control (Figure 1D). These results suggested that the virus entry processes would not stimulate DNA damage, and de novo viral protein expression and/or DNA replication may potentially account for this event. To test the hypothesis, virus infected MDBK cells were treated with phosphonoacetic acid (PAA), a specific inhibitor targeting viral DNA polymerase [34]. PAA at a concentration of 100 μM showed no cytotoxicity to MDBK cells (Figure 1H), but could reduce virus titer over 1log (Figure 1G). As expected, the generation of comet tails in the virus infected cells was obviously reduced by PAA treatment, in comparison with that in the mock treated control (Figure 1E). The tailDNA% in the virus infected cells (approximately 50%) was reduced to a level less than 30% by PAA treatment. Thereby, it seems that de novo viral protein expression and/or DNA replication correlated with the elevated levels of DNA damage.

### 3.2. The Involvement of ROS in BoHV-1-Induced DNA Damages in MDBK Cells 

**Figure 2:**
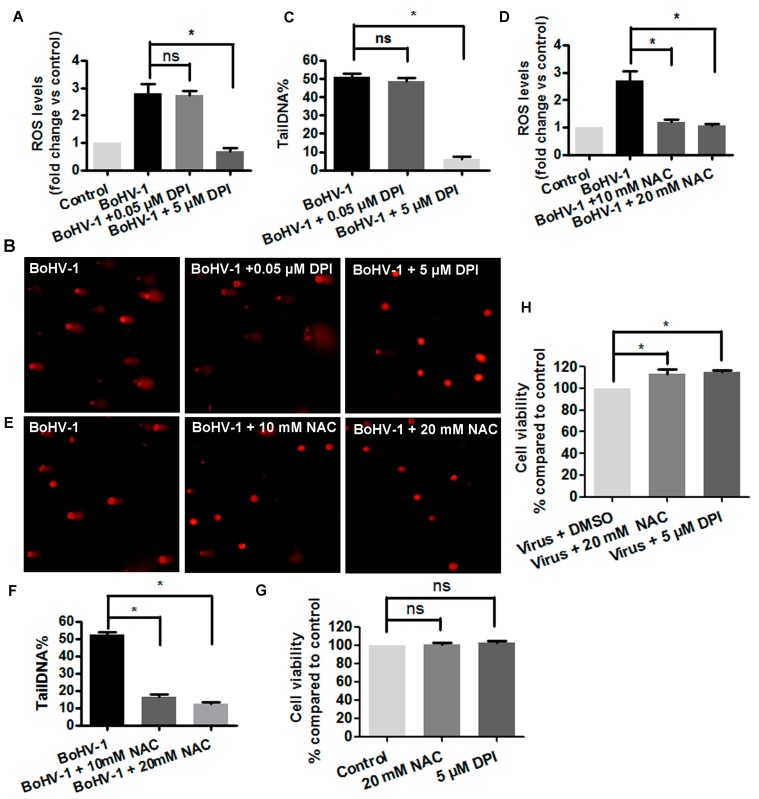
Reactive oxidative species (ROS) was involved in BoHV-1-induced DNA damage in MDBK cells. (**A**,**D**) MDBK cells were infected with BoHV-1 (MOI = 0.1) and treated with DPI (**A**) or NAC (**D**) at indicated concentrations. After infection for 24 h, cellular ROS levels were determined using H2DCFDA (5 μM, 30 min) (Sigma-Aldrich, St. Louis, MO, USA) and quantitatively analyzed using software Image-pro Plus 6. *, significant differences (*P* < 0.05), as determined by a Student *t* test. (**B**,**E**) MDBK cells were infected with BoHV-1 (MOI = 0.1), and treated with DPI (**B**) or NAC (**E**) at indicated concentrations. At 24 h after infection, DNA damage in individual cells was detected with comet assay, the images were acquired under a fluorescence microscope (Magnification ×200). (**C**,**F**) Three hundred cells were randomly selected from the samples treatment with either DPI (**C**) or NAC (**F**) for the analysis of TailDNA% with software CASP. *, significant differences (*P* < 0.05) in tailDNA%, as determined by a Student *t* test. (**G**) MDBK cells were treated with each chemicals at indicated concentrations for 24 h, and cell viability was evaluated by Trypan-blue exclusion test. Data represent the results from three independent experiments. Significance was assessed with the student *t* test (*, *P* < 0.05). (**H**) MDBK cells were infected with BoHV-1 (MOI = 0.1) and treated with either NAC or DPI at indicated concentrations for 24 h, and cell viability was evaluated by Trypan-blue exclusion test. Data represent the results from three independent experiments. Significance was assessed with the student *t* test (*, *P* < 0.05).

ROS are mainly generated by NADPH oxidases family of enzymes (NOXs). We have previously reported that both the NOXs inhibitor DPI and the ROS scavenger NAC could inhibit ROS production stimulated by BoHV-1infection in MDBK cells [35]. To test whether ROS regulates DNA damage in the context of virus infection, the cells were treated with either DPI or NAC. As a result, ROS levels were increased nearly 3 fold at 24 h after infection in MDBK cells, which was significantly decreased by the treatment with both DPI (5 μM) and NAC (10 and 20 μM), respectively (Figure 2A,D). As expected, the frequency of cells with comet tails were decreased by the treatment with either DPI (5 μM) or NAC (10 mM and 20 mM) (Figure 2B,E). Following the treatment by both DPI and NAC, tailDNA% showed a similar trend as that in ROS levels. When the virus infected cells were treated with 5 μM of DPI, the tailDNA% was decreased to 6.23%, while 0.05 μM of DPI had no effects (Figure 2C). Similarly, the treatment of virus infected cells by 10 mM and 20 mM of NAC, tailDNA% was decreased to 16.56% and 12.33%, respectively (Figure 2E,F). Trypan-blue exclusion test indicated that neither DPI (5 μM) nor NAC (20 mM) had obvious cytotoxicity to MDBK cells (Figure 2G), and they could efficiently rescue the virus infection-induced cell death, with cell viability increasing by approximately 15% (Figure 2H), which corroborated with our previous results that either DPI or NAC could significantly reduce virus productive infection [35]. Though both DPI and NAC may have off target effects, and block ROS with distinct mechanisms, they could unanimously inhibit virus infection-induced DNA damage, suggesting that ROS was involved in the generation of DNA damage. 

### 3.3. BoHV-1 Infection of MDBK Cells Promotes 8-oxoG Production

**Figure 3:**
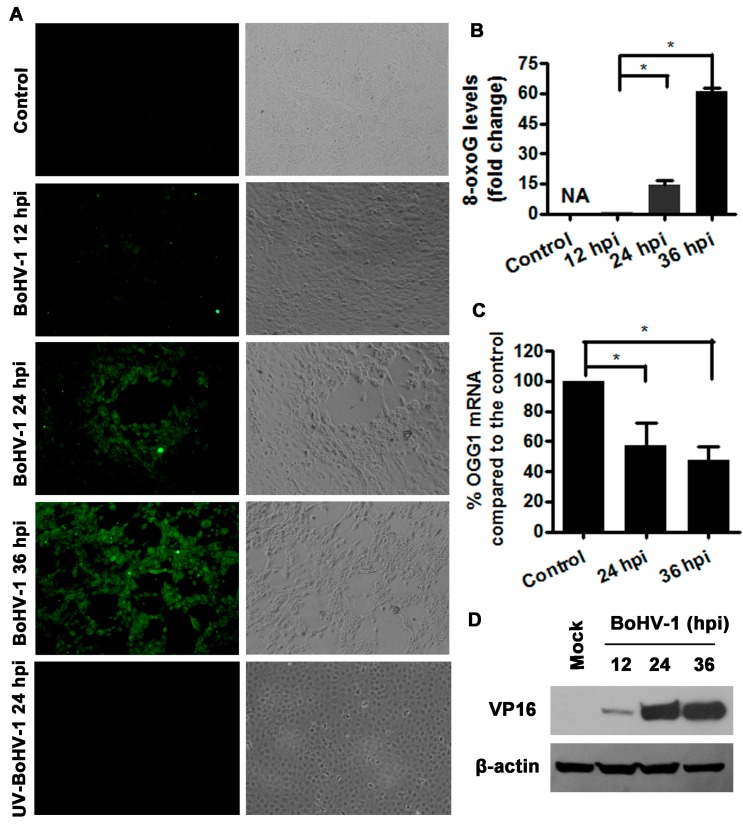
BoHV1 infection increased the levels of oxidative DNA damage marker 8-oxoG. (**A**) MDBK cells were infected by either BoHV-1 or UV-inactivated virus for designated periods. The production of 8-oxoG was detected with immunofluorescence assay. Images shown were represent of three independent experiments (Magnification ×200). (**B**) Fluorescence intensity of three images from each sample was analyzed with software image J. The relative fold change was calculated by normalization to the value of 12 h after infection which was arbitrarily set as 1. NA, not analyzed. (**C**) Total RNA was prepared at 24 and 36 h after infection in MDBK cells, and the mRNA levels of OGG1 were measured by qRT-PC. Each analysis was compared with that of uninfected control which was arbitrarily set as 100%. Data represent three independent experiments. Significance was assessed with the student *t* test (*, *P* < 0.05). (**D**) MDBK cells in 60 mm dishes were mock infected or infected with BoHV-1 (MOI = 0.1) for 12, 24 and 36 h. The cell lysates were prepared for Western blotting to detect the expression of viral protein VP16. Data shown are representative of three independent experiments.

8-oxo-7,8-dihydro-2-deoxyguanosine (8-oxoG), one of the most abundant form of oxidative DNA lesions is a widely used marker for oxidative stress-derived DNA damage [36]. To learn more about the potential for BoHV-1 infection induced oxidative DNA damage, we examined the influence of virus infection on 8-oxoG production in MDBK cells. The cells were infected with BoHV-1 for 12, 24 and 36 h, then immunoflouresence assay was performed using an antibody against 8-oxo-G. At 24 h after infection, sporadic cytopathic effect (CPE) foci with cell globularization was observed. Immunostaining positive cells were mainly observed in the CPE foci, but not in the areas without CPE. At 36 h following infection, extensive CPE was observed in the cell culture, and 8-oxoG immunostaining positive cells were extensively detected (Figure 3A). In view of the fact that the immunostaining in the mock infected control was too faint to be exactly assessed with software, it was not further subjected to quantitative analysis, and the values for 12 h after infection were used for normalization. After infection for 24 and 36 h, the levels of 8-oxoG production increased approximately 15 and 60-fold, respectively, relative to that at 12 h after infection (Figure 3B), suggesting that the 8-oxoG levels were increased during virus productive infection. In addition, we found that the UV-inactivated virus lost the ability to stimulate 8-oxoG production (Figure 3A). So, these results indicated that viral infection enhanced the production of 8-oxoG.

8-oxoG, induced by oxidative DNA damage, is mainly repaired by an enzyme, DNA glycosylase (OGG1) [37,38,39]. We then examined the effects of BoHV-1 productive infection on OGG1 expression by detection of OGG1 mRNA levels with qRT-PCR. As a result, virus infection led to a consistent decrease of OGG1 mRNA levels at 24 and 36 h after infection. Relative to the uninfected control, OGG1 mRNA levels decreased to 57.5% and 47.9% at 24 and 36 h after infection, respectively (Figure 3C). In addition, we detected the expression of viral protein VP16 to verify virus infection kinetics. VP16 can be readily detected at 24 and 36 h after infection, while a faint band can be detected only by overexposure at 12 h after infection(Figure 3D). These results indicated that BoHV-1 infection suppressed OGG1 mRNA expression, which was correlated with 8-oxoG accumulation. 

### 3.4. ROS Levels Are Increased in BoHV-1 Infected U2OS Cells and A549 Cells 

**Figure 4:**
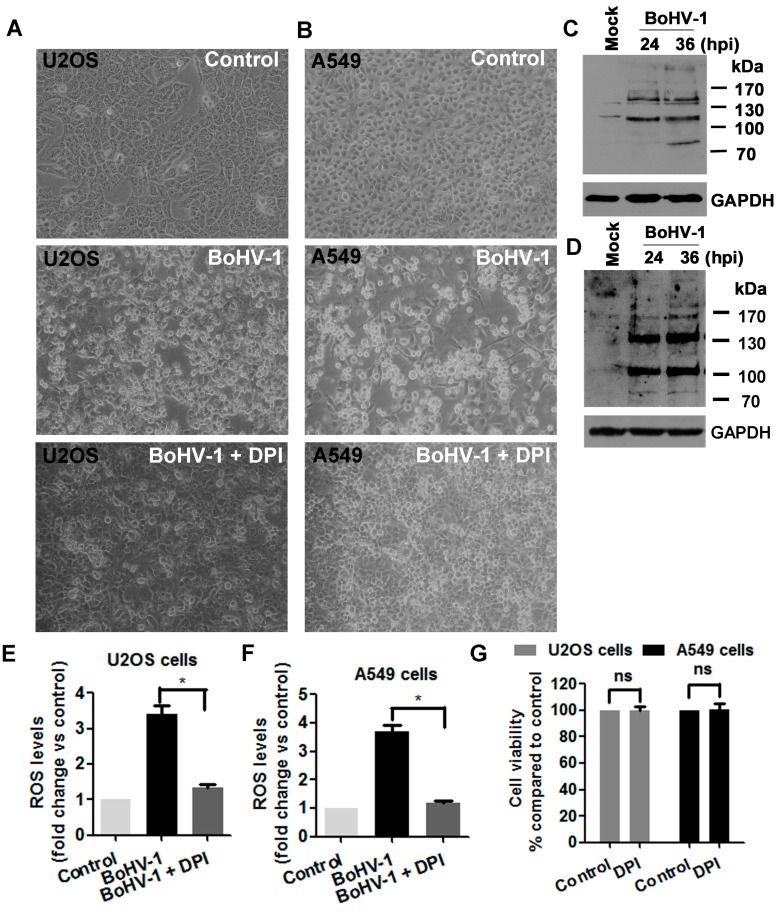
Virus infection enhanced ROS production during the infection of human tumor cells. (**A**,**B**) U2OS cells (**A**) and A549 cells (**B**) were infected by BoHV-1 (MOI = 0.1) for 48 h, with or without DPI (5 Μm) treatment. The cell morphology was observed under light microscope. Images shown were representative of three independent experiments (Magnification ×200). (**C**,**D**) U2OS cells (**C**) and A549 cells (**D**) were infected with BoHV-1 (MOI = 0.1). At 24 h and 36 h after infection, the cells lysates were prepared and subjected to Western blotting analysis using goat anti-BoHV-1 serum (VMDR Inc., 1:6000) to detect the expression of virion-associated proteins. Images shown were representative of three independent experiments. (**E**,**F**) U2OS cells (**E**) and A549 cells (**F**) were infected with BoHV-1(MOI = 0.1), and treated by 5 μM of DPI. After 24 h infection, cellular ROS levels were determined using H2DCFDA (5 μM, 30 min) and quantitatively analyzed using software Image-pro Plus 6. Results are means of three independent experiments. Significance was assessed with the student *t* test (*, *P* < 0.05). (**G**) U2OS cells and A549 cells were infected with BoHV-1 (MOI = 0.1) and treated by 5 μM of DPI. After infection for 48 h the cell viability was determined by Trypan-blue exclusion test. Data represent three independent experiments. Significance was assessed with the student *t* test (*, *P* < 0.05).

The cancer cell line U2OS originated from human osteosarcoma. A549 cells are adenocarcinomic human alveolar epithelial cells. BoHV-1 is a novel candidate oncolytic virus targeting numerous tumor cells of different origins [10]. According to the literature, BoHV-1 can infect both U2OS cells and A549 cells, and kill these tumor cells [10]. Here, our study further confirmed that the virus indeed can infect these cancer cells and induce CPE with distinct levels as representatively demonstrated at 48 h after infection (Figure 4A,B). In addition, the expression of certain virion-associated proteins in both cell cultures was detected by Western blots using an antibody against BoHV-1 virions (Figure 4C,D), though we could not point to the identity of these viral protein bands. These results are consistent with previous reports that the virus could infect these human tumor cells and induce cell death [10]. 

ROS overproduction induced by BoHV-1infection in neuronal and glial-derived tumor cells has been reported [17]. Here, we further explored the generation of ROS in the virus infected human osteosarcoma cells U2OS and human adenocarcinomic alveolar epithelial cells A549. As a result, virus infection in both U2OS cells and A549 cells stimulated an overproduction of ROS, which was increased more than three fold, as compared to that in the uninfected conrol, which decreased to a level close to that in the uninfected control by DPI treatment (Figure 4E,F). In addtion, the treatment by DPI (5 μM) obviousely prevented the the generation of CPE (Figure 4A). Of note, 5 μM of DPI did not show apparent cytotoxicity to either U2OS cells or A549 cells (Figure 4G). Therefore, our data suggested that BoHV-1 infection in U2OS cells and A549 cells stimulated ROS production.

### 3.5. BoHV-1 Infection Leads to Elevated DNA Damage in Human Tumor Cells Such as U2OS Cells and A549 Cells

**Figure 5:**
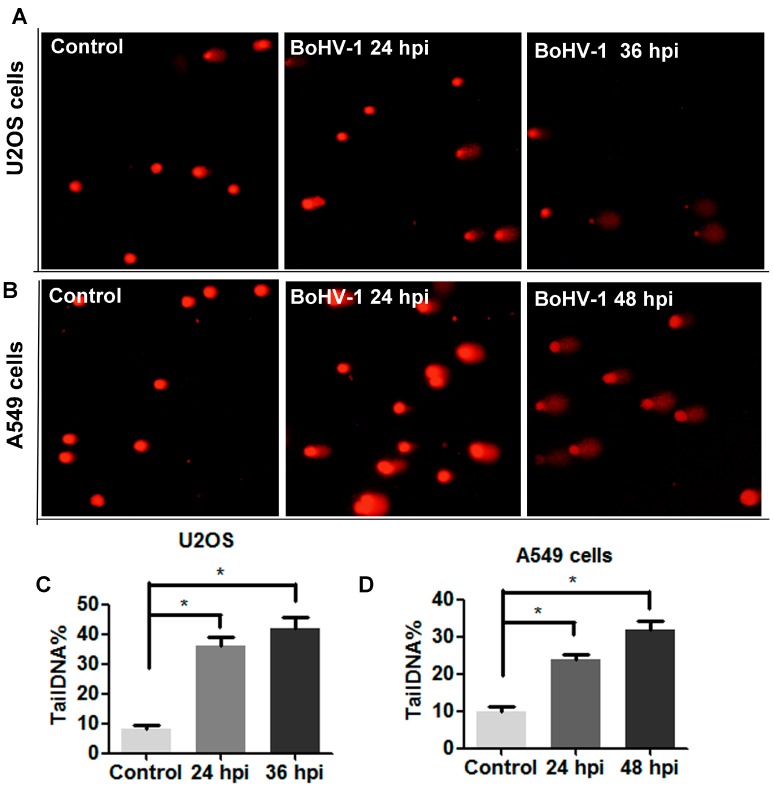
The detection of DNA damage in BoHV-1 infected human tumor cells. (**A**,**B**) Both U2OS cells (**A**) and A549 cells (**B**) were infected with BoHV-1 (MOI = 0.1) for designated times. The cells were collected and subjected to comet assay. Images shown were represents of three independent experiments (Magnification ×200). (**C**,**D**) Three hundred cells from either U2OS cells (**C**) or A549 cells (**D**) were randomly selected and calculated tailDNA% with software CASP. Values represented three independent experiments. (*, *P* < 0.05), significant differences was determined by a Student *t* test.

Since the findings described above confirmed that BoHV-1 can infect U2OS cells and A549 cells (Figure 4A,C), we wondered whether DNA damage was induced during the infection process. To address this question, comet assay was performed to assess DNA damage in both cells. The comet tails were readily observed in both cells following the virus infection at all the detected time points, but only a few positive cells were observed in the uninfected control (Figure 5A,B). The tailDNA% for the results shown in U2OS cells were: uninfected control as 8.1%, at 24 h after infection as 36.1%, and at 36 h after infection as 42.1% (Figure 5C). In A549 cells, tailDNA% were: uninfected control as 9.9%, at 24 h after infection as 23.8%, and at 48 h after infection as 31.8% (Figure 5D). Thus, these results indicated that BoHV-1 infection induced DNA damage in human tumor cells of both U2OS and A549 cells. 

### 3.6. ROS Is Involved in BoHV-1-Induced DNA Damage in Human Tumor Cells

**Figure 6:**
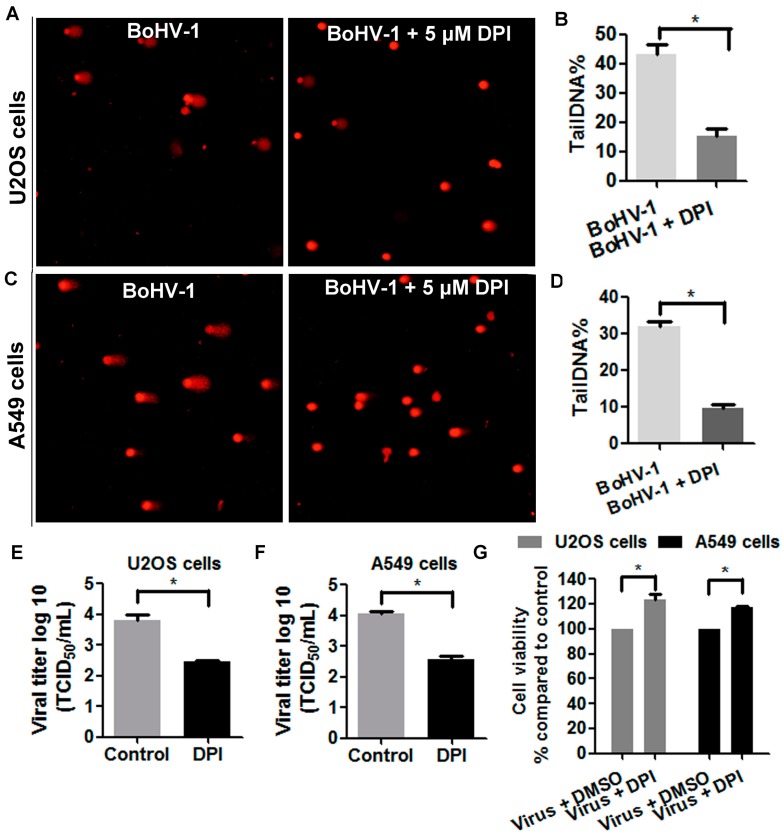
ROS was involved in BoHV-1-induced DNA damage in human tumor cells. (**A**,**C**) Both U2OS cells (**A**) and A549 cells (**C**) were infected with BoHV-1 (MOI = 0.1) and treated with DPI (5 μM) or DMSO control. At 48 h after infection, DNA damage in individual cells was detected with comet assay. The images shown represent three independent experiments (Magnification ×200). (**B**,**D**) Three hundred cells from both U2OS cells (**B**) and A549 cells (**D**) treated with or without DPI were randomly selected for the analysis of tailDNA% with software CASP. Results are means of three independent experiments. *, Significant differences *(P* < 0.05) in tailDNA%, as determined by a Student *t* test. (**E**,**F**) Both U2OS cells (**E**) and A549 cells (**F**) were infected with BoHV-1 (MOI = 0.1) and treated with or without DPI (5 μM). At 48 h after infection, the virus titers were detected using MDBK cells. *, significant differences *(P* < 0.05), as determined by a Student *t* test. (**G**) Both U2OS cells and A549 cells were infected with BoHV-1 (MOI = 0.1) and treated with DPI (5 μM) or DMSO control. At 48 h after infection, the cell viability was determined by Trypan-blue exclusion test. Results are means of three independent experiments. *, Significant differences *(P* < 0.05), as determined by a Student *t* test.

Since the findings described above demonstrated that the levels of both ROS and DNA damage were elevated in the virus infected U2OS cells and A549 cells, we examined whether ROS was involved in the generation of DNA damage. As shown in Figure 6A, at 48 h after infection comet tails were readily detected in a subset of mock treated infected U2OS cells, while only a few positive cells were detected in the cell cultures treated with 5 μM of DPI. The tailDNA% as shown in U2OS cells were: mock treated control as 43.1%, and treatment with 5 μM of DPI as 15.2% (Figure 6B). Similarly, the elimination of DNA damage by DPI was observed in virus infected A549 cells (Figure 6C). The tailDNA% as shown in A549 cells were: mock treated control as 43.1%, and treatment with 5 μM of DPI as 9.6% (Figure 6D). The decreased levels of tailDNA% in both U2OS cells and A549 cells by NOX inhibitor DPI suggested that ROS was involved in BoHV-1-induced DNA damage during the infection of these human tumor cells. Furthermore, we noticed that the treatment by 5 μM of DPI significantly affected the virus production, with virus titer decreased approximately 1.5 log in both cell cultures (Figure 6E,F), and prevented the cells from death, with cell viability increasd approximately 20% in both cell cultures (Figure 6G). These results consistently added support to our hypothesis that oxidative DNA damage may potentially contribute to the virus-induced cell damage, which is a potential mechanism for the concolytic effects. 

## 4. Discussion 

Generally, DNA is under constant threat from multiple sources, including errors during DNA replication, products of intrinsic cellular reactions (e.g., ROS) and environmental factors such as UV radiation and chemical exposure [40]. Therefore, DNA damage is a common event in the life of a cell and may lead to mutation, cancer, or tissue death [41]. It has been elucidated that multiple viruses’ infection increase DNA damage, which is implicated in the disease progress. For example, hepatitis C virus promotes hepatocarcinogenesis development through the manipulation of DNA damage [42], and oxidative DNA damage is induced by influenza virus-induced inflammation, which may contribute to cytotoxicity in vivo [43]. In addition, damage to DNA may stimulate a series of pathways referred to as DNA damage response (DDR) signaling that enables the cells either to eliminate or to activate a programmed cell death process, and drives inflammatory responses [41,44]. It seems that DNA damage due to infection is an important cellular factor potentially associated with virus pathogenesis. In this study, for the first time, we revealed that BoHV-1 infection of bovine kidney cells induced DNA damage (Figure 1), and that the inhibition of ROS production by either NAC or DPI could rescue the virus infection-induced DNA damage and cell death (Figure 2). Importantly, during our preparation of this manuscript, a paper has been published revealing that BoHV-1 tegument protein VP8 induces cell apoptosis through blocking the DDR proteins including ataxia telangiectasia mutated (ATM), phosphorylates Nijmegen breakage syndrome (NBS1) and structural maintenance of chromosome-1 (SMC1), which may consequently lead to the inhibition of DNA repair [45]. Thus, it is reasonable to speculate that the induction of DNA damage correlates with BoHV-1-caused cell damage.

The inhibition of BoHV-1-induced DNA damage by both DPI and NAC suggested that the generation of DNA damage was partially associated with ROS production, which is not surprising because the virus infection stimulates ROS overproduction which would potentially assault DNA. In addition, two independent studies have demonstrated that BoHV-1 infection stimulates ROS overproduction that leads to mitochondria dysfunction in neuronal and glial-derived tumor cells, and MDBK cells [17,35]. Here, the finding that ROS contributed to BoHV-1-induced DNA damage would highlight the cellular toxicitity of overproduced ROS during virus infection, which may further address the importance of ROS for the viral pathogenecity. 

BoHV-1is an attractive candidate for oncolytic virotherapy of tumors originated from multiple tissues. However, how BoHV-1 infection exerts its oncolytic effects remained unclear. In this study, for the first time we demonstrated that BoHV-1 infection of human tumor cells such as in U2OS cells and A549 cells induced oxidative DNA damage, which is a potential mechanism by which the virus kills tumor cells because DNA damage is a toxic signaling that could trigger mitochondria-dependent and -independent death machineries. Apart from U2OS cells and A549 cells identified in this study, DNA damage may be induced during the virus infection of the other tumor cells, which need further investigation. Interestingly, accumulated studies have highlighted the potential of targeting DNA damage and DDR signaling as a promising therapeutic strategy for the treatment of multiple cancers [46,47]. For example, Olaparib approved by the FDA as a treatment for metastatic breast cancer in 2018, works maingly through its DNA-damaging effects [47]. In addition, 5-Azacytidine, a DNA methyltransferase inhibitor, and temozolomide, an alkylating agent, widely used in cancer therapy are also functionally depending on the induction of DNA damage [48,49,50]. Obviously, just like these chemotherapeutic drugs, BoHV-1 may take advantage of the cellular DNA-damage machinery to kill tumor cells, which further address the potential of this virus as a promising virotherapy. 

The combination of oncolytic herpes simplex virus (G47Δ) and temozolomide acted synergistically in killing glioblastoma stem cells (GSCs) [51]. Though the mechanisms underlying the synergistic anticancer effects are not clear, it is obvious that DNA damage is involved in the process because temozolomide could induce DNA damage and high levels of DNA damage were really detected after combination treatment. Similarly, BoHV-1 and 5-Azacytidine combination improved the therapeutic efficacy in a tolerized cotton rat model of breast adenocarcinoma [11]. Given that both BoHV-1 and 5-Azacytidine could induce DNA damage, it is reasonable to speculate that induction of robust DNA damage may contribute to the enhanced therapeutic outcome. Therefore, our study may provide a possible perspective to explain the oncolytic effects of BoHV-1 used along or in combination with the other drugs such as 5-Azacytidine. In addition, it may be implicated to inform further development of novel combination therapy strategies to combat cancer.

## 5. Conclusions 

In summary, we provided evidence that BoHV-1 infection induced DNA damage in both MDBK cells and human tumor cells, a potential mechanism by which the virus infection lead to cell damage. This finding might add to our knowledge and understanding, not only the viral pathogenesis, but also the virus oncolytic effects that are important for development of novel cancer therapy strategies.
